# Outcomes of T-cell lymphoblastic lymphoma in children and adolescents treated with Dana-Farber Cancer Institute Childhood ALL Consortium protocols

**DOI:** 10.3389/fped.2025.1686081

**Published:** 2026-01-12

**Authors:** Giacomo Gotti, Yael Flamand, Victoria Koch, Sabrina Testa, Kristen Stevenson, Thai-Hoa Tran, Bruno Michon, Uma Athale, Lewis B. Silverman, Yana Pikman, Andrew E. Place

**Affiliations:** 1 Department of Pediatrics, Fondazione IRCCS San Gerardo dei Tintori, Monza, Italy; 2 Department of Data Science, Dana-Farber Cancer Institute, Boston, MA, United States; 3 Dana-Farber/Boston Children’s Cancer and Blood Disorders Center and Harvard Medical School, Boston, MA, United States; 4 Department of Biostatistics and Computational Biology, Dana-Farber Cancer Institute, Boston, MA, United States; 5 Division of Pediatric Hematology-Oncology, Charles-Bruneau Cancer Center, CHU Sainte-Justine, Université de Montréal, Montréal, QC, Canada; 6 Division of Pediatric Hematology-Oncology, CHU de Quebec, Saint-Foy, QC, Canada; 7 Division of Hematology Oncology, McMaster Children’s Hospital, and Department of Pediatrics, McMaster University, Hamilton, ON, Canada; 8 Division of Pediatric Hematology, Oncology and Stem Cell Transplantation, Columbia University Medical Center, New York, NY, United States

**Keywords:** Lymphoblastic lymphoma, treatment protocols, outcomes, T-cell lymphoblastic lymphoma, prognostic factors

## Abstract

Treatment approaches to childhood T-cell lymphoblastic lymphoma (T-LL) are based on those used for T-cell acute lymphoblastic leukemia (T-ALL), but reports of outcomes with contemporary regimens are limited, as patients with LL are often excluded from ALL clinical trials. In this study, we retrospectively analyzed the characteristics and outcome of a cohort of 23 pediatric patients with T-LL treated between 2006 and 2020 according to Dana-Farber Cancer Institute (DFCI) ALL Consortium protocols. Five-year event-free survival, overall survival, and disease-free survival rates were 78.3% (95% CI: 55.4%–90.3%), 87.0% (95% CI: 64.8%–95.6%), and 90% (95% CI: 65.6%–97.4%), respectively. Morphological marrow disease (defined as 5%–24% blasts) at diagnosis was the only feature associated with adverse prognosis. Treatment based on DFCI ALL protocols is an effective strategy for childhood LL and should be considered at the time of treatment selection.

## Introduction

Lymphoblastic lymphoma (LL) accounts for approximately 20% of childhood non-Hodgkin lymphoma (1), with the majority characterized by T-cell immunophenotype, arising from immature lymphocytes. Grouped together in the 2022 World Health Organization classification (2), LL differs from acute lymphoblastic leukemia (ALL) in the extent of marrow involvement (absent or <25% lymphoblasts in LL) (3).

The outcome of pediatric patients with LL has dramatically improved with the adoption of ALL-based regimens based on the Berlin–Frankfurt–Müenster (BFM) ALL chemotherapy backbone. The NHL-BFM90 trial reported a 5-year event-free survival (EFS) rate for T-cell LL (T-LL) of 90% (4), but a similar result was not achieved in the following NHL-BFM95 and EURO-LB02 trials, reporting a 5-year EFS of 82% for T-LL (5, 6). Favorable outcomes were obtained for T-LL patients treated on the COG AALL0434 trial, in which the 4-year EFS and overall survival (OS) rates were 84.7% and 85.9%, similar to those obtained for T-ALL patients treated in the same study (7). The efficacy of other pediatric regimens for T-LL has not been extensively reported (8, 9). In this study, we describe the characteristics and outcomes of a series of children and adolescents with T-LL treated according to Dana-Farber Cancer Institute (DFCI) ALL Consortium regimens.

## Methods

Patients diagnosed with T-LL between 2006 and 2020 treated according to a DFCI ALL Consortium protocol were included in the study. LL was diagnosed by biopsy or cytology of involved site and bone marrow evaluation demonstrating <25% lymphoblasts. Morphologic bone marrow involvement was defined by the presence of ≥5% lymphoblasts. Minimal bone marrow involvement was defined as being less than 5% of marrow lymphoblasts detected using flow cytometry and/or fluorescence *in situ* hybridization (FISH). Central nervous system (CNS) status was defined as CNS1 (absence of blasts and <5 leukocytes/µL), CNS2 (presence of blasts and <5 leukocytes/µL), or CNS3 (presence of lymphoblasts and ≥5 leukocytes/µL, cranial nerve palsies, or identification of leukemic infiltrates of the leptomeninges or brain parenchyma) based on initial diagnostic evaluation.

Patients were classified as high risk (HR) because of the T-lineage or as very high risk (VHR) based on the presence of adverse cytogenetics such as a *KMT2A* rearrangement, same as that for patients with T-ALL treated on DFCI ALL Consortium trials (10). Unlike for ALL, end-induction minimal residual disease (MRD) was not included in the risk stratification process.

Patients diagnosed between 2006 and 2011 (*n* = 7) were treated according to the DFCI 05-001 (NCT00400946) protocol and patients diagnosed after 2012 (*n* = 16) were treated according to DFCI 11-001, of whom 6 were enrolled on the multicenter DFCI 11-001 study (NCT01574274) (11, 12). These two protocols shared the same multiagent chemotherapy backbone (Supplementary Table S1) but differed in the use of cranial radiation and asparaginase randomization. All T-LL patients treated according to DFCI 05-001 received cranial radiation. For patients treated according to DFCI 11-001, cranial radiation was restricted only to those who presented with CNS3 disease or who were classified as VHR. Patients who had CNS2 or CNS3 disease at diagnosis received additional doses of intrathecal chemotherapy during remission induction on both protocols.

Complete remission (CR) assessment was performed on Day 32 of induction and defined as a reduction ≥70% in the size of the largest nodes or masses noted at diagnosis by computed tomography or positron emission tomography, with concomitant marrow morphologic remission and absence of CNS disease. EFS was defined as the time from diagnosis to the time of induction failure, induction death, and relapse or death due to any cause and censored at the time last known alive and event free. Induction failures and induction deaths were considered events at time zero. OS was defined as the time from diagnosis to death from any cause and censored at the time last known alive. Disease-free survival (DFS) was defined as the time from achievement of CR to the time of relapse or death in continuous CR. EFS, DFS, and OS were estimated using the Kaplan–Meier method.

## Results

The clinical, disease, and treatment characteristics of the cohort of 23 patients are summarized in Table 1. The majority of patients (20/23, 87%) presented with a mediastinal mass. Five patients (22%) had morphologic bone marrow involvement and 11 (48%) had minimal bone marrow involvement. Four patients (17%) had CNS2 and one had CNS3 disease, and all five were treated with additional lumbar punctures during induction; all of these patients had minimal or morphologic marrow involvement.

**Table 1:** Patient and treatment characteristics.

Characteristics	N	%
Number of patients	23	100
Age (years)		
<10	10	44
≥10	13	56
Median age, years (range)	11.5 (3.6–17.2)	
Sex		
Male	19	87
Female	4	13
Mediastinal mass		
Yes	20	83
No	3	17
CNS status		
CNS1	18	78
CNS2	4	17
CNS3	1	4
Bone marrow involvement		
Morphologic (≥5%)	5	22
Minimal (<5%)	11	48
None	7	30
Status at end induction		
CR	20	87
ID	1	4
IF	2	9
Risk group (*n* = 20)		
HR	19	95
VHR	1	5
Cranial radiation (*n* = 20)		
No	13	65
Prophylactic	6	30
Therapeutic	1	5

CNS, central nervous system; CR, complete remission; ID, induction death; IF, induction failure; HR, high risk; VHR, very high risk.

Genetic abnormalities were found in 11 patients (48%) by karyotype or FISH analysis of the mass biopsy or bone marrow aspirate, and these are reported in Table 2. The final risk group was assigned to patients achieving CR at the end of induction. Among them, all patients were classified as HR because of T-cell immunophenotype and one was classified as VHR because of the presence of a *KMT2A* rearrangement. Four patients received dexamethasone (6 mg/m^2^/day) during induction instead of prednisone because of physician choice, and two patients received an intensified consolidation I phase for the persistence of residual disease or in response to high end-induction MRD detected in the bone marrow.

**Table 2:** Cytogenetic abnormalities, treatment, and outcome characteristics of T-LL patients.

Patient	Site for karyotype analysis	Karyotype	FISH results	Initial BM involvement	Risk group	Treatment protocol	Event	Outcome
1	BM	46,XY	Positive for 12p gain^a^	No	HR	05-001	No	Alive CCR
2	BM	46,XY	Negative^b^	No	HR	05-001	No	Alive CCR
3	BM	46,XY	Positive for 12p deletion	Morphologic	NA	05-001	IF	Alive CCR
4	BM	46,XY	ND	Minimal	HR	05-001	No	Alive CCR
5	BM	46,XY	ND	No	HR	05-001	No	Alive CCR
6	BM	46,XY	Positive for 9p21 deletion	Morphologic	NA	05-001	ID	Dead
7	EM	47,XX,+r	ND	Minimal	HR	05-001	No	Alive CCR
8	EM	46,XY	ND	No	HR	11-001	No	Alive CCR
9	EM	No metaphases	ND	No	HR	11-001	No	Alive CCR
10	EM	46,XY	Positive for 9p21 deletion	No	HR	11-001	No	Alive CCR
11	BM	46,XY	Negative^b^	Minimal	HR	11-001	No	Alive CCR
12	EM	47,XX,del(3)(p21p23),+8,t(9;16)(p22;q12-13),t(11;19)(q23;p13.3)	Positive for 11q23 rearrangement	Minimal	VHR	11-001	No	Alive CCR
13	EM	No metaphases	ND	Morphologic	HR	11-001	Relapse	Dead
14	BM	46,XY	Negative^b^	Minimal	HR	11-001	No	Alive CCR
15	EM	45,XY,dup(6)(p21.1p25),add(7)(q22),add(7)(q32),der(9;17)(q10;q10)	Positive for 9p21 deletion	Minimal	HR	11-001	No	Alive CCR
16	EM	46,XY	Positive for 9p21 deletion^c^	Minimal	HR	11-001	No	Alive CCR
17	BM	46,XY	Positive for 9p21 deletion	Minimal	HR	11-001	No	Alive CCR
18	EM	46,XY	Positive for 14q11.2 rearrangement	Minimal	HR	11-001	No	Alive CCR
19	BM	46,XX	Negative^b^	No	HR	11-001	No	Alive CCR
20	BM	47,XX,+4, der(12)t(1;12)(p22;q24.3)	Positive for trisomy 4	Morphologic	HR	11-001	No	Alive CCR
21	EM	46,XY	Negative^b^	Minimal	HR	11-001	No	Alive CCR
22	BM	46,XY	Negative^b^	Minimal	NA	11-001	IF	Dead
23	BM	46,XY	ND	Morphologic	HR	11-001	Relapse	Alive

BM, bone marrow; CCR, continuous complete remission; EM, extramedullary; HR, high risk; ID, induction death; IF, induction failure; NA, not applicable; ND, not done; VHR, very high risk.

a FISH performed on pleural effusion.

b FISH negative for *BCR::ABL1*, *ETV6::RUNX1*, and *KMT2A* rearrangement.

c FISH performed on BM.

All five patients treated as per the DFCI 05-001 protocol had CNS1 status and received 12 Gy prophylactic cranial radiation. Only 2 of the 15 patients treated as per the DFCI 11-001 protocol were irradiated because of the VHR risk group (12 Gy) and CNS3 status at diagnosis (18 Gy). Patients did not receive radiation to the mediastinum or other sites of disease.

**Figure 1:**
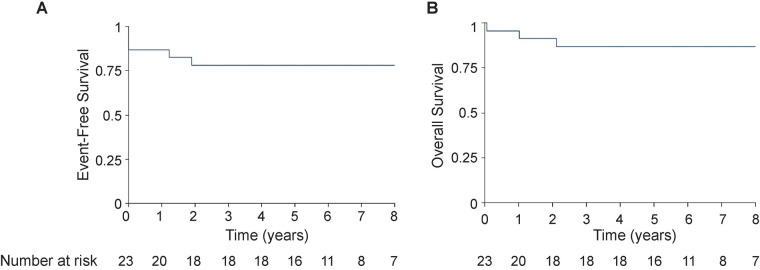
Event-free survival (**A**) and overall survival (**B**) at 8 years.

CR was achieved in 20 (90%) patients, of whom 11 had complete radiologic response and 9 had a residual mass but ≥70% reduction in size compared with baseline. Overall, five events were observed. One patient died during induction because of trauma-induced intracranial hemorrhage. Two patients were classified as having induction failure; one had persistent morphologic bone marrow disease at the end of induction but subsequently achieved remission after reinduction and was proceeded to undergo allogeneic transplantation, and the other had a persistent mediastinal mass at the end of induction and ultimately died because of refractory disease after multiple lines of therapy were initiated. Two other patients experienced CNS relapse while on treatment and achieved remission after reinduction; one is still alive after allogeneic hematopoietic stem cell transplantation and the other died because of experiencing infection while in remission. Four of five events occurred in patients who presented with ≥5% marrow blasts at diagnosis. Notably, four of six patients who presented with Murphy Stage IV disease experienced an event. There were no events in nine patients who met the protocol definition of CR but still had a radiologic evidence of a residual mass. Overall, 18 of 23 patients with T-LL achieved CR and remained event free at the time of analysis. No events occurred in patients who received cranial radiation. Events by protocol are reported in Supplementary Table S2. The median follow-up time was 6.9 years (range 0.07–11.8). The 5-year EFS and OS were 78.3% (95% CI: 55.4%–90.3%) and 87% (95% CI: 64.8%–95.6%), respectively (Figure 1). The 5-year DFS was 90% (95% CI: 65.6%–97.4%).

## Discussion

For T-ALL and T-LL, a lack of effective salvage regimens mandates the use of effective upfront risk stratification and therapies. We report a retrospective series of 23 pediatric patients with T-LL treated according to DFCI ALL Consortium protocols. Our cohort had baseline clinical characteristics similar to those reported in the literature. Overall outcomes [5-year EFS of 78.3% (95% CI: 55.4%–90.3%) and 5-year OS 87.0% (95% CI: 64.8%–95.6%)] were comparable to outcome results reported for patients with T-LL treated with other regimens (6–8, 13).

One of the challenges in LL treatment is the identification of reliable prognostic factors useful for risk-adapted therapy, and a few studies have investigated whether the presence of minimal marrow disease at diagnosis or radiologic criteria for early response influences outcome (14, 15). An analysis of 86 patients with T-LL enrolled in the COG AALL1231 study revealed end-induction MRD measured by flow cytometry at <0.1% to be associated with a superior 4-year EFS, although without an impact on OS (16). It is not clear whether more sensitive methods of MRD detection or different time points would allow for improved prognostication (17, 18). In addition, recent studies have included a deep genetic characterization of T-ALL and T-LL to identify the predictors of relapse (19, 20) and inform differences between T-ALL and T-LL, but strong predictors are lacking. In our series, 70% of patients had evidence of marrow involvement at diagnosis and four out of five events occurred in patients with morphologic marrow involvement at diagnosis. No relapses were observed in nine patients who had a residual mass at the end of induction but otherwise met CR criteria (≥70% shrinkage of the mass in size). With technological advances, future studies will need to integrate T-LL genomics, more precise bone marrow disease detection using next-generation sequencing, MRD, and possibly other factors for achieving a more precise prognostication. Given the rarity of this disease, multicenter collaborative trials will be needed.

Our patients were treated with conventional chemotherapy using HR or VHR therapy arms of DFCI ALL protocols. Compared with the COG AALL1231 strategy, our approach does not include a delayed intensification phase, but patients receive a prolonged administration of pegaspargase in addition to anthracyclines as part of the postinduction treatment (Supplementary Table S1). Given the poor outcome of patients with relapsed/refractory disease, the introduction of novel therapies in the frontline treatment has been investigated. The COG AALL1231 study demonstrated the efficacy of bortezomib in patients with T-LL but not for patients with T-ALL (21). In contrast, the COG AALL0434 study showed that the addition of nelarabine significantly improved the outcomes of patients with T-ALL but not those with T-LL (7).

As adopted by most cooperative groups (5, 22), treatment intensity reduced with the omission of prophylactic cranial radiation for all T-LL patients in the DFCI 11-001 protocol, without a significant increase in the relapse rate. The outcomes of patients with T-LL treated with the DFCI ALL approach were similar to those reported by other groups (4, 6, 7, 21). All patients were treated using the HR or VHR treatment arms of the respective protocols. Future studies should focus on identifying patients who may be successfully treated with less intensive regimens. Beyond its retrospective nature, our report is limited by the small sample size, which affects the strength of our findings and the identification of prognostic risk factors.

In conclusion, our retrospective analysis reports on the efficacy of the DFCI ALL Consortium-based approach for the treatment of T-LL (23). Future studies should focus on the identification of prognostic variables and integration of novel therapies to improve outcomes and minimize the long-term toxicity of treatment.

## Data Availability

The raw data supporting the conclusions of this article will be made available by the authors without undue reservation.
