# Supplementary material for: Outcomes of T-cell lymphoblastic lymphoma in children and adolescents treated with Dana-Farber Cancer Institute Childhood ALL Consortium protocols
Source: Front Pediatr. 2026 Jan 12;13:1686081. doi: 10.3389/fped.2025.1686081 (PMC12833020; doi:10.3389/fped.2025.1686081)
Supplement: Supplementary file 1 [file Table1.docx]

Supplementary Table 1. Treatment Plans for DFCI ALL Consortium Protocol 11-001 High and Very High Risk Arms

| **Phase** | **Treatment Details** |
| --- | --- |
| Remission induction  duration 32 days | *Steroid Prophase (Days 1-3)*  MPDN 32 mg/m^2^/day  *Remission Induction Therapy (Days 4-32)*  VCR 1.5 mg/m^2^ (max 2 mg) IV, days 4, 11, 18, 25  PRED 40 mg/m^2^/day PO, days 4-32 (followed by taper over < 7 days)  DOX 30 mg/m^2^/dose IV, days 4-5 with dexrazoxane 300 mg/m^2^/dose IV  MTX 40 mg/m^2^ IV push, day 6  PEG-ASP 2,500 IU/m^2^ IV, day 7  IT-cytarabine dosed by age, day 1^a^;  IT-MAH dosed by age, day 18;  IT-MTX dosed by age, day 32 |
| Consolidation I  HR: duration 3 weeks  VHR: duration 9 weeks | *Consolidation IA*  VCR 2 mg/m^2^ (max 2 mg) IV, day 1  6-MP 50 mg/m^2^/day PO, days 1-14  HD-MTX 5 g/m^2^ IV over 24 hours, day 1, followed by LCV rescue  IT-MTX dosed by age, day 1^b^  DOX 30 mg/m^2^/dose IV, day 1 with dexrazoxane 300 mg/m^2^/dose IV  *VHR patients receive two additional cycles:*  *Consolidation IB*  CP 1,000 mg/m^2^ IV, day 1  Cytarabine 75 mg/m^2^/day IV, days 2-5, 9-12  6-MP 50 mg/m^2^/day PO, days 1-14  IT-MTX dosed by age, day 1  *Consolidation IC*  Cytarabine 2 g/m^2^/dose IV every 12 hours (4 doses), days 1-2  VP-16 100 mg/m^2^/dose IV daily, days 3-5  DEX 18 mg/m^2^/day PO or IV twice daily, days 1-5  PEG-ASP 2,500 UI/m^2^, beginning on day 8 every 2 weeks (15 doses)^c^ |
| CNS phase  duration 3 weeks | *All patients:*  VCR 2 mg/m^2^ (max 2 mg) IV, day 1  6-MP 50 mg/m^2^/day PO, days 1-14  DEX 18 mg/m^2^/day PO twice daily, days 1-5  DOX 30 mg/m^2^/dose IV, day 1 with dexrazoxane 300 mg/m^2^/dose IV  IT-MAH dosed by age, twice weekly starting on day 1 (4 doses)  *HR patients:*  PEG-ASP 2,500 UI/m^2^ IV, starting day 1^c^  Cranial radiation (only for those CNS-3 at diagnosis and VHR patients) |
| Consolidation II  duration 27-30 weeks^d^ | *All patients receive every 3-week cycles of:*  VCR 2 mg/m^2^ (max 2 mg) IV, day 1  6-MP 50 mg/m^2^/day PO, days 1-14  DEX 18 mg/m^2^/day PO twice daily, days 1-5  IT-MAH, on day 1 every 9 weeks for 6 doses and then every 18 weeks  DOX 30 mg/m^2^/dose IV, day 1 with dexrazoxane 300 mg/m^2^/dose IV^e^  PEG-ASP 2,500 UI/m^2^ IV every 2 weeks to complete 15 postinduction doses. |
| Continuation  duration approximately 71 weeks^f^ | *All patients receive every 3-week cycles of:*  VCR 2 mg/m^2^ (max 2 mg) IV, day 1  6-MP 50 mg/m^2^/day PO, days 1-14  DEX 6 mg/m^2^/day PO twice daily, days 1-5  MTX 30 mg/m^2^ IV or IM weekly, days 1, 8, 15 (week 1 MTX held if IT-MTX given)  IT-MAH on day 1 every 9 weeks for 6 doses and then every 18 weeks^g^ |

Abbreviations: 6-MP, 6-mercaptopurine; ALL, acute lymphoblastic leukemia; CSF, cerebrospinal fluid; CNS, central nervous system; CP, cyclophosphamide; CR, complete remission; DEX, dexamethasone; DFCI, Dana-Farber Cancer Institute; DOX, doxorubicin; HD-MTX, high-dose methotrexate; HR, high risk; IM, intramuscular; IT, intrathecal; IT-MAH, IT methotrexate/cytarabine/hydrocortisone; IV, intravenously; LCV, leucovorin; MPDN, methylprednisolone; MTX, methotrexate; PEG-ASP, pegaspargase pegol; PRED, prednisone; PO, orally; VCR, vincristine; VHR, very high risk; VP-16, etoposide.

^a^ Patients with CNS leukemia at diagnosis (CNS-2 and CNS-3) received IT-cytarabine twice weekly until CSF was clear of blasts cells on 3 consecutive examinations

^b^ Not administered if high-dose–MTX started within 72 hours of IT-MTX given on day 32 of induction.

^c^ Pegaspargase 2,500 IU/m^2^/dose IV every 2 weeks (15 total doses). Continues at designated interval across treatment phases through consolidation II once started (within CNS phase for HR patients, within consolidation IC for VHR patients)

^d^ Consolidation II ends when patient has achieved 30 weeks of PEG-ASP therapy and has reached cumulative DOX dose of 300 mg/m^2^

^e^ Once cumulative DOX reaches 300 mg/m^2^, weekly MTX is substituted at 30 mg/m^2^ IV or IM, days 1, 8, and 15

^f^ Continuation therapy discontinued after 104 weeks of continuous CR

^g^ IT-MAH administered every 18 weeks in Consolidation II and Continuation phases for those who received previous cranial radiation
