# Supplementary material for: Outcomes of T-cell lymphoblastic lymphoma in children and adolescents treated with Dana-Farber Cancer Institute Childhood ALL Consortium protocols
Source: Front Pediatr. 2026 Jan 12;13:1686081. doi: 10.3389/fped.2025.1686081 (PMC12833020; doi:10.3389/fped.2025.1686081)
Supplement: Supplementary file 2 [file Table2.docx]

Supplementary Table 2. Events of study cohort overall and by treatment protocol

|  | **Overall cohort** | **DFCI 05-001 cohort** | **DFCI 11-001 cohort** |
| --- | --- | --- | --- |
| Number of patients | 23 | 7 | 16 |
| CR at end of induction | 20 | 5 | 15 |
| Induction failure | 2 | 1 | 1 |
| Induction death | 1 | 1 | 0 |
| Relapse | 2 | 0 | 2 |

Abbreviations: CR, complete remission; DFCI, Dana-Farber Cancer Institute
